# A case requiring re-thoracotomy due to a significant reduction of tidal volume after commencement of chest tube drainage under pressure control ventilation following lower lobectomy

**DOI:** 10.1186/s40981-022-00526-3

**Published:** 2022-05-24

**Authors:** Taichi Shiraishi, Shinju Obara, Takahiro Hakozaki, Tsuyoshi Isosu, Satoki Inoue

**Affiliations:** Department of Anesthesiology and Division of Intensive Care, Fukushima Medical University, 1 Hikarigaoka, Fukushima, 960-1295 Japan

**Keywords:** Chest drainage, Transpulmonary pressure, Pressure-controlled ventilation

## Abstract

**Background:**

The use of pressure-controlled ventilation (PCV) during one lung ventilation (OLV) has been popular to avoid high airway pressure. We experienced a case of a significant reduction of tidal volume (TV) after commencement of chest tube drainage under PCV following lower lobectomy, which required re-thoracotomy to evaluate the degree of air leak.

**Case presentation:**

A 70-year-old man was scheduled for a lower lobectomy. OLV was managed by PCV. The driving pressure was set at 15–20 cmH_2_O with 4 cmH_2_O of positive end-expiratory pressure (PEEP). A chest drainage tube was placed after completion of lobectomy. To switch OLV to two lung ventilation (TLV), PCV settings were changed to the driving pressure at 10 cmH_2_O with 4 cmH_2_O of PEEP, which generated 450 ml of TV. Immediately after applying drainage (−10 cmH_2_O), TV decreased down to 250 ml. To maintain 450 ml of TV, PCV was switched to volume-controlled ventilation with 450 ml of TV, which raised the plateau pressure close to 24 cmH_2_O. Re-thoracotomy was done; however, significant findings were not detected.

**Conclusions:**

We experienced a case of a significant reduction of TV immediately after chest tube drainage following lower lobectomy. Probably, negative intrapleural pressure increased the residual volume, which might have significantly affected the limited lung volume after lobectomy, resulting in decreasing TV during PCV.

## Background

The use of pressure-controlled ventilation (PCV) during one lung ventilation (OLV) has been popular to avoid high airway pressure [1]. A survey showed that the majority of survey respondents favor PCV over volume control ventilation (VCV) [2]. However, any definitive conclusions regarding which is better for OLV, PCV, or VCV have not yet been made [1]. Chest tube placement is a common procedure after lung surgery to drain fluid, blood, or air from the pleural cavity. Negative pressure is usually applied to the chest drainage system to generate negative pressure in the intrapleural space. Therefore, the negative pressure generated by the drainage system might affect transpulmonary pressure (TPP), which is defined as the difference between alveolar and intrapleural pressure [3]. We experienced a case of a significant reduction of tidal volume (TV) after commencement of chest tube drainage under PCV following lower lobectomy, which required re-thoracotomy to evaluate the degree of air leak.

## Case presentation

Written informed consent was obtained from the patient for publication of this case report and accompanying images. A 70-year-old man (height 176 cm, weight 53 kg) was admitted to our hospital for the removal of suspected lung cancer lesions in the left lower lobe. He was suffered from mild hypertension and diabetes mellitus and was a past smoker. His preoperative pulmonary function test showed that percent vital capacity was 111% and percent forced expiratory volume in 1 s was 65.4%. Severe adhesion of the interlobar fissure was suspected. Thus, a thoracotomy was scheduled.

A para-midline thoracic epidural puncture was performed in the left lateral position at the T6/T7 interspinous space using an 18 G Tuohy epidural needle. General anesthesia was induced with target controlled infusion (TCI) of propofol set at 3 μg/mL plus remifentanil 0.3 μg/kg/min, and neuromuscular blockade was achieved with 50 mg of rocuronium. Tracheal intubation with a left-sided 37 Fr double lumen tube (DLT) was placed for OLV and the correct position was confirmed by auscultation and fiberoptic bronchoscopy. General anesthesia was maintained with 2.5–3 μg/mL of TCI propofol, 0.2–0.3 μg/kg/min of remifentanil, epidural bolus injection of 4–5 ml/h of 0.375% ropivacaine and 10–15 mg/h of rocuronium. Aestiva/5 7900 (GE Healthcare Japan, Tokyo) was used for respiratory management during anesthesia. OLV was managed by PCV. The driving pressure was set at 15–20 cmH_2_O with 4 cmH_2_O of positive end-expiratory pressure (PEEP) to maintain 5–6 mL/kg of TV, the respiratory rate was adjusted to maintain PaCO_2_ at around 40–45 mmHg, and FiO_2_ was adjusted to maintain SpO_2_ more than 95%.

The left lower lobectomy was uneventfully performed in the right lateral position and air leak was checked several times by saline submersion test with a peak pressure of 15–20 cmH_2_O. Every time an air leak was detected, parenchymal suturing was performed until the air leakage became self-limiting. A chest drainage tube was placed at the 6th–7th intercostal space at the level of the midclavicular line. Immediately after closure of the chest wall, OLV was switched to two lung ventilation (TLV) and the driving pressure of PCV was changed to 10 cmH_2_O with 4 cmH_2_O of PEEP, which generated 450 ml of TV.

**Fig. 1:**
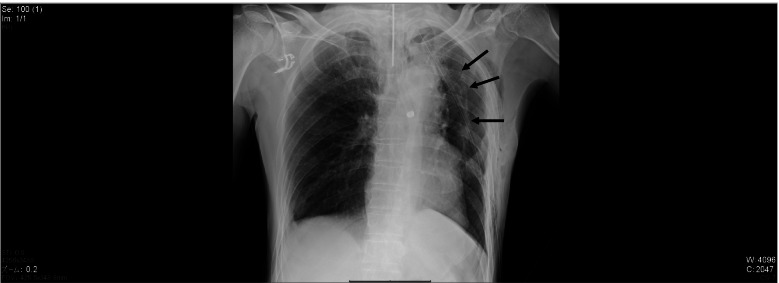
Postoperative chest X-ray image. The volume of the left lung was reduced. The allows indicate the visceral pleura

After confirming a steady state of ventilation, the chest tube drainage was started at −10 cmH_2_O. Immediately after starting drainage, a low TV alarm occurred, indicating a decrease in TV down to 250 ml. To maintain 450 ml of TV, PCV was switched to VCV with 450 ml of TV, which raised the plateau pressure close to 24 cmH_2_O. Simultaneously, the chest tube drainage system demonstrated significant air leakage. DLT position was reconfirmed by fiberoptic bronchoscopy. After discussion about suture failure with thoracic surgeons, we decided to evaluate suture failure under re-thoracotomy. However, the air leakage checked by saline submersion test was still self-limiting. After the chest wall was closed, TLV was restarted with the same PCV settings, which achieved 450 ml of TV. Immediately after the restart of the chest tube drainage, TV again decreased to 250 ml; however, the air leakage was not significant. We judged that there was no severe air leak. Therefore, we increased the driving pressure up to 20 cmH_2_O to generate 450 ml of TV. After the patient’s position was changed to supine, TV increased to 800–850 ml at the same PCV settings. The postoperative chest X-ray image was unremarkable (Fig. 1). Soon after, his trachea was extubated. His postoperative course was uneventful.

## Discussion

**Fig. 2:**
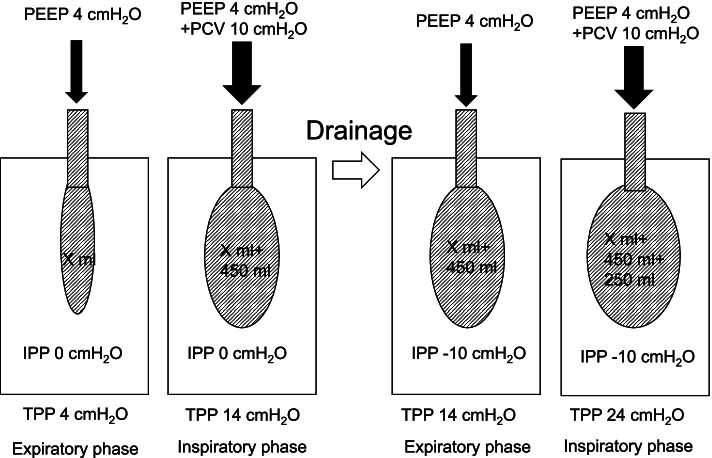
The phenomenon happened after drainage. The left side of the figure shows the lung state during PCV without drainage. The right side of the figure shows the lung state under PCV after starting drainage. After drainage of – 10 cmH_2_O, TPP became 14 cmH_2_O at the expiratory phase, which would have generated X + 450 ml of residual volume. Here, 10 cmH_2_O of driving pressure increased TPP to 24 cmH_2_O but only generated 250 ml of TV probably because of increased elastance by overextension of the lung. PEEP, positive end-expiratory pressure; Plateau Prs, plateau pressure; *PCV*, pressure control ventilation; *VCV*, volume control ventilation; *IPP*, intrapleural pressure; *TPP*, transpulmonary pressure

**Fig. 3:**
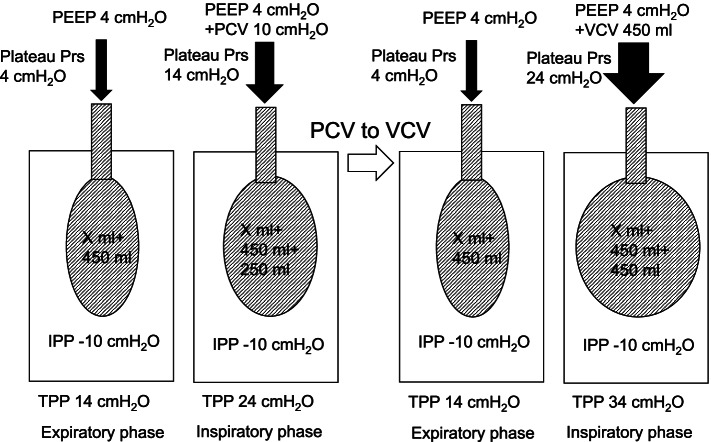
The phenomenon happened after switching pressure control ventilation to volume control ventilation. The left side of the figure shows the lung state during PCV with drainage. The right side of the figure shows the lung state after switching PCV to VCV. In case of VCV, 450 ml of TV forced lung volume to increase from X + 450 ml to X + 450 + 450 ml, which increased plateau pressure to 24 cmH_2_O because of more increased elastance by more overextension. *PEEP*, positive end-expiratory pressure; *Plateau Prs,* plateau pressure; *PCV*, pressure control ventilation; *VCV*, volume control ventilation; *IPP*, intrapleural pressure; *TPP*, transpulmonary pressure

This uncommonly observed event was probably caused by an increase in TPP due to the chest tube drainage system. Before drainage, assuming that there were X ml of residual volume with 4 cmH_2_O of PEEP (TPP = 4 cmH_2_O) at the expiratory phase, 10 cmH_2_O (TPP = 14 cm H_2_O) of driving pressure during PCV generated 450 ml of TV (Fig. 2, left). After drainage of – 10 cmH_2_O, TPP became 14 cmH_2_O at the expiratory phase, which would have generated X + 450 ml of residual volume. Here, 10 cmH_2_O of driving pressure increased TPP to 24 cmH_2_O but only generated 250 ml of TV probably because of increased elastance by overextension of the lung (Fig. 2, right). In case of VCV, 450 ml of TV forced lung volume to increase from X + 450 ml to X + 450 + 450 ml, which increased plateau pressure to 24 cmH_2_O because of more increased elastance by more overextension (Fig. 3, right). It was calculated that TPP at the inspiratory phase would be 34 cmH_2_O at that time. However, these suggested mechanisms cannot go beyond speculation because we did not measure TPP. Furthermore, since this explanation is not based on an artificial physiological model, we may give careful consideration to a concern that the suggested phenomenon could occur in the actual cases. In short, this phenomenon would require special conditions. For example, in cases with pleural adhesion or inhomogeneous lesions in the lungs, it is not certain whether negative pressure can be equally delivered to the whole lung. Therefore, in such cases, the theoretical TTP might not be applied to the entire part of the remaining lung as it was.

We assume that the phenomenon occurred in a very specific situation because our speculation was based on the case that ventilation was maintained only by the ipsilateral lung. At that time, ventilation was maintained by two lungs. And, the contralateral lung should not have been affected by the drainage system. In the present case, considering the TV increase in the supine position, the contralateral lung might not have well functioned after the restart of TLV in the lateral position for some reasons and ventilation might have depended on the ipsilateral lung. It has been suggested that lung elastance is increased in the lateral position with one-lung ventilation [4]. Therefore, the ventilation state of the contralateral lung might have changed in the lateral position. In such a situation, large volume resection of the lung was considered to significantly affect the elastance of the residual lung, which might have limited TV by PCV. Actually, residual lung volume was apparently reduced on the postoperative chest X-ray image (Fig. 1). Negative intrapleural pressure by drainage system increased TPP at the expiratory phase and was equal to the driving pressure of PCV. Therefore, the residual volume with the drainage system was equal to the lung volume at the inspiratory phase without the drainage system. Thus, the residual volume increased by TPP generated due to negative intrapleural pressure might have significantly affected the limited lung volume after lobectomy.

The observed air leakage before re-thoracotomy was maybe due to the residual large volume of air in the pleural space because the large volume resection of the lung should have left a large dead space. This explains why we did not detect a large volume of air leakage after almost completely draining the air. The large volume of air leakage and a concern of the possibility of suture failure because of intraoperative extensive adhesiotomy let us perform re-thoracotomy. However, considering that we obtained the same expiratory volume as the set TV at VCV, we should have judged that there was little air leakage even though PCV volume decreased at the same settings. Otherwise, it would have been much better if we could have checked the inspiratory and expiratory volume simultaneously on the spirometry display; however, the display we used could not show the inspiratory volume during PCV mode. Or, if we had closely checked whether plateau pressure could be maintained at the level of the set driving pressure with PEEP, we could have determined whether there was significant air leakage or not. Lastly, although PCV is commonly used during OLV, we keep in mind that this phenomenon could only occur due to PCV settings.

In conclusion, we experienced a case of a significant reduction of TV after commencement of chest tube drainage under PCV following lower lobectomy. Probably, negative intrapleural pressure increased the residual volume, which might have significantly affected the limited lung volume after lobectomy, resulting in decreasing TV at PCV. The phenomenon we experienced might be rare and only occur in a specific situation; however, it is necessary to make a right decision whether the leakage is fake or real. Besides, we should keep in mind that negative intrapleural pressure generated by chest tube drainage could significantly affect respiratory condition against our expectation.

## Data Availability

Not applicable

## Abbreviations

PCV: Pressure-controlled ventilation
OLV: One lung ventilation
VCV: Volume control ventilation
TPP: Transpulmonary pressure
TV: Tidal volume
TCI: Target controlled infusion
DLT: Double lumen tube
PEEP: Positive end expiratory pressure
Plateau Prs: Plateau pressure
